# Motivating change in resident language use through narrative medicine workshops

**DOI:** 10.1186/s12909-022-03721-z

**Published:** 2022-09-07

**Authors:** Kristin Collier, Amit Gupta, Alexandra Vinson

**Affiliations:** 1 University of Michigan, Michigan, USA; 2 University of North Carolina at Chapel Hill, North Carolina, USA

**Keywords:** Narrative medicine, Doctor-patient communication, Medical training

## Abstract

**Background:**

There are many ways that students and trainees learn to talk about patients. The way trainees and physicians use language during clinical care is important, as labeling patients can have adverse effects on patient safety. Communication is considered a core competency by The Accreditation Council on Graduate Medical Education (ACGME). Past research has shown that participants in narrative medicine curricula report developing stronger communication skills however it is not clear how these workshops motivated trainees to use language differently during patient care. To explore this, we interviewed second-year residents in academic year 19–20 about their experiences both in participating in narrative medicine workshops and giving patient care.

**Methods:**

The framing context for this constructivist thematic analysis is a series of narrative medicine workshops facilitated for interns in an internal medicine residency program at a large academic medical center during the 18–19 academic year. We developed a semi-structured interview study that allowed residents to reflect on their experiences in these workshops. Eighteen out of 60 residents (30%) were interviewed.

**Results:**

We found that sessions regarding language use in patient care shaped how interns thought about and used language during clinical work, a finding that arose spontaneously during interviews.

**Conclusions:**

Our research suggests that workshops aimed specifically at addressing the use of language in healthcare can have meaningful impact on trainees. Our study makes a unique contribution to the scholarship by suggesting that training in narrative medicine can lead to a change in the way that trainees use language during their clinical work.

## Introduction

Language matters in patient care. It is the tool by which we communicate with our patients, our colleagues and even ourselves. As Charon writes, there are four central narratives that exist in healthcare [1]. The first narrative is the story that the patient tells their healthcare provider about their illness. The second type of narrative is the story we tell other providers – either in a formal presentation on rounds, in a handoff during a transition of care, or to a colleague for a quick curbside consult. The third narrative is the one we have with ourselves. As meaning making beings we are constantly trying to make sense of what we witness in medicine through the running narrative we have in our mind about the patients we’ve seen and the stories they’ve told us. And lastly, some of us tell patient stories to society at large – in advocacy work, op-ed writing, and publications.

There are many ways in which students and trainees learn to talk about patients – through the formal curriculum, role modeling, and the hidden curriculum. All of these forces shape trainees’ learning on the wards, in the clinics, and in the classroom. Hafferty and Franks described the overall process of medical education as a form of “moral training” of which the formal curriculum is only a small part [2]. The hidden curriculum “deals with the tacit ways in which knowledge and behavior are constructed” that operates in parallel with the formal, explicit curriculum of medical training [3]. How our trainees learn to talk about patients is shaped by this hidden curriculum.

How we learn to use language in our everyday lives is greatly influenced by the culture which surrounds us. The culture of healthcare, like any culture, has conventions of language use—its members use language in patterned and orderly ways [4, 5]. One reason why it is important to study language use in patient care—and to train physicians to be reflexive about their own language use—is that the language we use in patient care affects the way we see our patients, the care they receive and even our ability to connect with them [6–8]. As Charon writes, the “care of the sick unfolds in stories” [9]. However, the processes by which trainees learn medical language and professional language conventions can be very subtle. For example, sociologist Howard Becker described his experience when the medical team in which he was embedded used the word “crock” to describe a patient. He came to realize how many meanings were built into that one word, as it was used for someone the medical team didn’t like, who took up too much time and who didn’t have “legitimate” concerns [10].

The way trainees and physicians use language as part of clinical care has raised concerns because certain types of language, including terms such as ‘gomer,’ ‘vegetable,’ and ‘circling the drain’ may have negative implications for patient and provider well-being [8]. Labeling patients can stigmatize them, delegitimize their concerns, and adversely affect patient safety [11]. Dehumanizing language can also be detrimental for staff. When healthcare workers start to see their patients as diseases, or even as non-persons, the humanism in the encounter can be stripped away, which may put healthcare workers at increased risk for burnout [12]. This language is common not only in verbal communication but in charting as well [13].

Many reasons have been hypothesized as to why healthcare workers use dehumanizing language. One reason is that this type of language serves as a coping mechanism by providing some “emotional distance” from the patient [7]. Using language that detaches the healthcare workers from the humanity of the patient therefore helps them to cope with the difficult situations they encounter in their work [14]. Dehumanizing language often reflects an underlying bias on the part of healthcare professionals against certain members of society, such as incarcerated persons [15]. Certain “favorable” patients are less likely to have such language used in their care. For example, patients who are seen by healthcare workers as caring about their health and participating in their care are less likely to be targets of derogatory language [16]. In sum, language use influences the culture of medicine and therefore the care of its patients.

Because of the salience of language use in patient care, there have been efforts to incorporate clinical skills training into medical education at the undergraduate medical education (UME) and graduate medical education (GME) levels. The Accreditation Council on Graduate Medical Education (ACGME) has also identified communication as a core competency and many patient satisfaction questionnaires include questions on the way that their healthcare provider communicated with them [17]. In recent decades, communication skills have frequently been taught and assessed using simulated patients, direct observation and feedback, and role plays with debriefing [18–21]. However, as Artfield and colleagues write, “these methods may create a false sense of measurement when applied to nuanced, context-based behaviors, and may encourage students to ‘perform’ exterior actions rather than function through critically examined internal attitudes” [22]. In other words, these exercises can feel like play-acting, raising concerns that while trainees may be able to perform in needed ways during clinical encounters, they may not develop deeper humanistic attitudes toward patient care. Moreover, recent concerns have been expressed that trainees may experience phenomena like empathic dissonance, “the mental discomfort created by the act of making expressions of empathy that are not sincerely felt” [23].

Because of these concerns, many schools and programs have developed and utilized training in narrative medicine for their learners. Narrative medicine, as defined by Charon, is medicine that is practiced with “narrative skills of recognizing, absorbing, interpreting, and being moved by the stories of illness” [24]. It is a discipline through which healthcare providers learn to attend to the psychological and relational aspects of patients’ experience with illness using story. In one study, students who participated in a narrative medicine curriculum reported an “enhanced understanding of and capability in communication” because of their training and said they had enhanced development of specific communication skills such as self-awareness, articulation, observation, patience, and empathy [22]. In another study, participation by medical professionals in a narrative medicine program resulted in increased self-reported empathy scores that persisted over time [23].

Thus, past research has shown that participants in narrative medicine curricula report developing stronger interpersonal communication skills and greater attention to their own communication practices. However, it is not clear how these workshops may have motivated trainees to use language differently during patient care and team communication. To explore this, we interviewed 18 second-year residents about their experiences participating in narrative medicine workshops and their experiences giving patient care. We found that sessions regarding language use in patient care shaped how interns thought about and used language in their interaction with their colleagues and their patients, a finding that arose spontaneously during interviews.

## Methods

### Background

The framing context for this study is a series of narrative medicine workshops facilitated for interns (first-year residents) in an internal medicine residency program at a large academic medical center. The workshops were part of a larger narrative medicine curriculum that was created by [Author 1, Author 2] and others for the interns over the year. The sessions were held at a protected time that was set aside weekly for didactics, but attendance was not mandatory.

The narrative medicine sessions covered a variety of topics. Topics covered included rhetoric and discourse communities, medical errors and the practice of forgiveness, imposter syndrome and mental wellness, “VIP syndrome” and health equity, reflective storytelling, and language in critical illness. The sessions were facilitated by a moderator and involved a combination of close reading, active listening, writing and reflection. We received consultation on the development of our sessions from experts in Narrative Medicine both nationally and locally and [Author 1] received training in narrative medicine through a workshop with Dr. Charon’s team at Columbia University. Two of the sessions specifically focused on the use of language in patient care. The first of these was on rhetoric and discourse communities and was led by one of our senior medical residents who had a background in the humanities and in creative writing. A discourse community is a group of individuals who share a common way of communicating about their shared values, goals and mission. The concept of discourse communities was introduced, with the goal of helping the participants to understand the type and nature of language used in medicine and how this differs from language used by other professions and even lay people. Workshop participants discussed the goal of using such language and the pitfalls thereof. The participants were then showed famous one-liners from works of fiction to illustrate how much can be inferred from one line of text. Then, one-liners from patient charts were displayed to reinforce the idea that through shared conventions of language use in medicine, much can be conveyed to the reader, with very few words. The use of words that label patients as their disease was discussed as well as the potential harm in doing so. The second language focused session was led by faculty from the division of pulmonary and critical care. Participants read and discussed excerpts from “In Shock” by Dr. Rana Awdish and “You can Stop Humming now” by Dr. Daniela Lamas, focusing on common phrases used in the intensive care unit (ICU), such as “circling the drain,” that can serve to dehumanize and objectify patients. Time for reflection was spent trying to understand why this language is used as well as how to better humanize the ICU experience for patients and providers alike.

### Study design

We developed a semi-structured interview study that would allow residents to reflect on their experiences of the Narrative Medicine workshops and, more broadly, about their experiences with patient care. The study was reviewed by the IRB at [our institution] and classified as exempt research.

### Study sample and recruitment

All second-year residents in Internal Medicine (*N* = 60, 32 men and 28 women, 52 internal medicine and 8 internal medicine-pediatrics) at [institution] were contacted via email and invited to participate in a brief semi-structured interview that could be held during work hours. Recruitment was conducted by [Author 2], who was a fellow at the same institution. In the case of no response, two follow-up emails were sent. Second year residents were also reminded of the opportunity to interview in a non-targeted manner by course facilitators while on their clinical rotations. At the onset of the COVID-19 pandemic, which overlapped with our recruitment period, an additional follow-up email was sent to all second year residents offering interview opportunities via Zoom. Ultimately, 18 out of 60 second year residents (30%, 10 men and 8 women, 17 internal medicine and 1 internal medicine-pediatrics) participated in an interview. While we hoped to interview as many residents as possible in order to gain the most comprehensive understanding of their experiences, we found that interviewing one third of the cohort did give us a sufficient understanding of their experiences. Moreover, considering the timing of our data collection overlapped with the onset of the COVID-19 pandemic, as we explain below, we were grateful that residents took time to speak with us.

Of these interviewees, 17 attended a language use workshop called “Discourse Communities” and 12 attended a language-oriented workshop called “Critical Illness.” These interviewees were able to speak to their experiences in these workshop sessions, their patient care experiences as residents, and reflect on how their participation in workshop sessions impacted their language use in patient care.

### Data collection

The interviews were conducted by [Author 2] and two additional study team members between December 2019 and June 2020. This time period included a data collection hiatus as the medical community responded to the onset of the COVID-19 pandemic. The interviews were collected face-to-face and via Zoom, as data collection became virtual due to human subjects research restrictions during the COVID-19 pandemic. While these interviews were brief, the interview guide included both open-ended questions and directive prompts designed to elicit specific recollections and experiences in a short time frame. The interview guide development was led by [Author 3], and explored areas of interest to our team, including motivation to attend narrative medicine workshops, salient memories from these workshops, clinical experiences that triggered recollections from the workshops, peer discussions about narrative medicine, experiences of burnout, and intent to participate in narrative medicine activities in the future. Participants provided verbal and written consent to participate in an interview and to have the interview recorded, and zero participants declined to have their interview recorded. Participants received no compensation for their participation. All three interviewers were post-graduate trainees at the same institution as the participants. This data collection strategy was designed to minimize power difference between workshop facilitators, including [Author 1], and interviewees.

### Data analysis

Interview recordings were transcribed using Rev.com and inductively coded using the Dedoose Platform (SocioCultural Research Consultants, Los Angeles, California) to generate and refine themes using a process of thematic analysis [25]. First, all authors independently open-coded transcripts to generate codes. Second, using a built-in coding agreement tool within Dedoose, one author coded a transcript with “test” codes. The other two authors were able to code duplicates of this transcript. Once the original codes were revealed, the authors were able to assess their level of agreement and reach consensus through discussion of areas of disagreement. We found a high level of agreement in our coding choices, and were able to proceed confidently with independent coding, using regular team meetings to discuss new codes and existing code application. Through discussion and immersion in the data, the authors constructed themes from the data set, and these themes were refined in the process of writing the paper.

## Findings

We identified three themes related to the topic of language use through the coding and refining process. These themes illustrate changes in how interviewees considered language use in clinical care in light of their participation in the workshops. The first theme, *Rising Awareness*, describes an increase in the interviewees’ awareness of the use of dehumanizing language in clinical care. We found that interviewees shared a new appreciation for the impact of “labeling patients.” In the second theme, *At the Bedside*, interviewees noted a higher level of awareness at the bedside that corresponded with a more contemplative stage characterized by increased self-reflection and introspection with regards to their own language choice. While the narrative medicine sessions were held apart from the clinical environment, many interviewees shared how they were reminded of the sessions when subsequently caring for patients at the bedside. Several interviewees focused on language use in the critical care setting and noted often catching their non-verbal thoughts and reframing those in a more humanizing manner because of their participation in the workshops. The third theme, *Intent to Change*, described the highest level of change, a change in one’s actions, in which interviewees described correcting their own language or planning to correct the language of others, as well as finding opportunities to course correct the habit formation of younger trainees. Multiple residents shared a deeper appreciation not only for their personal communication practices, but also for the impact these can have on patient care due to altered perceptions of patients by the medical community. Overall, interviewees reflected on their passive awareness of language use, the influence of the workshops on their active generation of new thoughts, and finally provide us insight into the process of reshaping their own communication practices. By gaining appreciation for the power of dehumanizing language, a few interviewees also reflected on their ability to be influencers of a cultural shift, especially with younger trainees and in communication with healthcare providers in other specialties.

### Rising awareness

Dehumanizing language is prevalent in healthcare but is often so much a part of the workplace that it may go unnoticed [13]. Because it is taken for granted in healthcare work, an important first step is becoming aware of one’s own language use and reflecting on the effects it may have. Residents who participated in narrative medicine workshops commented that they became more aware of the use of dehumanizing language in patient care. Sometimes this awareness developed during patient care and attracted residents to the workshop. In other cases, residents’ awareness was raised during the workshops.

In explaining this awareness during interviews, residents reflected on the reasons that such language may be used. For example, when asked about their motivation for attending the narrative medicine sessions, one interviewee described:

The one that I went to, the premise definitely spoke to me, that it's something that I had noticed before that we use some of this negative talk, and it feels comfortable in a way. It's kind of a way to build camaraderie, but also is kind of negative. I thought it would be interesting to hear what other people thought about it and to kind of delve into that more. (Man, Internal Medicine)

In this instance, the resident was previously aware of “negative talk,” and was curious to hear what others thought. This suggests that the narrative medicine workshop provided a setting to have a conversation with peers about a salient aspect of everyday work that was not occurring elsewhere. As one interviewee recalled:

I think one that I remember specifically, there was one part where they were talking specifically about the language being used in the ICU. Things like the types of terms and slang thrown around like circling the drain, or that sort of thing. I thought it was nicely done, and it was something that I haven't really heard addressed that way before. Because it's true, you throw these kinds of phrases around, and maybe it's in some way a form of dealing with the stress or to compartmentalize in a way. (Man, Internal Medicine)

In this case, the resident noted that the narrative medicine workshop addressed the issue of language use in a novel way that resonated with their own experience. This resident further speculated that using certain slang terms might be a way of dealing with the stress of working in the ICU, and hinted that they had personal experience with using such phrases during their own time in the ICU. The phrase ‘circling the drain’ is evocative, and was used by several interviewees. This is a phrase sometimes used by care teams to describe a patient whose death is imminent. In her book, *In Shock*, Dr. Rana Awdish describes her memory of hearing this phrase used about her when she was critically ill and remembers thinking “that statement could have been the last thing I ever heard” [26]. The phrase can be understood as inherently dehumanizing, as it brings to mind an image of something that is about to be discarded as trash, and as Dr. Awdish reports, may be overheard as a verdict by patients themselves. However, as one resident described, negative talk may also function as a form of self-protection that shifts the blame from physicians when a patient’s outlook is not good. For example, when asked at the beginning of the interview what they could recall from the narrative medicine sessions they attended, one interviewee said:

The one that I remember going to was, I believe, the last one that was about language that we use, especially in the ICU, but really, it's anywhere, to talk about patients and our experiences that... focusing on language that we use as protective, but may be negative in some way. So, specific phrases I remember discussing are things like circling the drain or... That's the one I can remember as sort of the example from the beginning, but I remember going through a lot of different kind of terminology that we use that can be used to kind of protect ourselves or place blame on someone else for a negative situation, not necessarily in a malicious way, but that was sort of the theme that I remember taking away, that there are a number of things that we say that are self-protective, but really are kind of negative, and we should be cautious when using those. (Man, Internal Medicine)

During interviews, residents theorized that much of the language used may be based in self-protective coping mechanisms: camaraderie with colleagues, compartmentalization of human emotions, management of stress, and displacement. Because the narrative medicine workshops gave residents an opportunity to focus on their own language use that they may not have otherwise had, they were able to discuss how such language may have negative impacts on the care team’s regard of the patient, even while it serves as self-protection for individual physicians. Residents described that the sessions made them more aware of the language they use and the effect such language can have with regards to internal perceptions of patients. They described this language as “negative,” implying that the use of such language can be dehumanizing and negatively impact one’s interactions and framing of a patient.

The use of dehumanizing language could also create an environment for propagation of stigma by labeling patients as not worthy of care. Respondents reported that participating in narrative medicine workshops raised their awareness of these issues, and that this translated into appreciation for the potential impact that language can have on individual patient interactions and more generally, patient care:

I remember he had just talked about how the terminology we use affects how we perceive patients. I think that some examples he used were like alcohol abuse, cirrhosis. What else did he talk about? Polysubstance use, IV drug use. And then, it was the first time that I think I had thought about how... You know, I think if you had asked me, I would have told you, "Yes, these terms carry a negative stigma," but I had never really thought about what that meant for patient care. So I do remember for me, it was thought provoking. (Man, Internal Medicine)

Participation in the workshops also helped residents orient to their own emotional reactions during patient care. As one resident noted:

I think in terms of the terminology. I think about that all the time. I'm looking up a chart, and I realize, "Oh, I'm having an emotional reaction to what I'm reading. I have to check my... Think about my bias." (Man, Internal Medicine)

This may have particular implications for residents who provide care to patients with complex needs who are often framed as “difficult”:

I had a patient who got referred to me from [a large urban referring hospital] where everything written about this patient was difficult patient, drug seeking, chronic pain, fibromyalgia, narcotic dependence, all these things. And leading up to the session, I was like, "Oh, great. I'm excited for what patient I'm about to get.” And then I thought about this like, "You know what? I need to reframe this because it's not going to help me have a good session with this patient and it's not going to help them get any better healthcare." (Man, Internal Medicine)

Here, our findings show that participation in the narrative medicine workshops helped residents understand communication practices in clinical care and raised their awareness of their own practices and how they may contribute to stigma and disparities in care. Beyond thinking of their language use as an individual phenomenon, participants in the workshops consistently reported on language use as an aspect of medical culture. Indeed, one resident commented on the phenomenon of ‘contagion’ and how the culture within the practice of critical care is shaped by the language that is used:

And I thought it was interesting how they brought it up, especially the effects that can be had from using that type of language and it's kind of contagious in a way and forms a culture in the ICU of that way of looking at people. (Man, Internal Medicine)

By participating in the workshops, residents became more aware of the use of dehumanizing language, reasons for its use, and an awareness of how using “negative talk” or stigmatizing language might adversely affect patient care. Respondents also highlighted the broadly shared patterns of language use, particularly in critical care teams. When language use is patterned and informally perpetuated through everyday medical work, it may create a norm that is difficult to counteract. For this reason, we were interested to explore how workshop participants may have used language differently while providing care and how they may have prompted new standards of language use on their teams.

### At the bedside

Often in medical training, there are “disconnections between the worlds of medical education and practice” whereby things learned in a didactic or ‘formal’ part of the curriculum are not applied at the bedside [27]. In our interviews, however, residents recalled how what they learned in the sessions came to mind when they were caring for patients at the bedside.

Yeah. I mean, was a little bit more cognizant of the patient. If we had conversations in the room to talk about them as a person, and not in like a third-person type of deal or an abstract kind of way, didn't really use some of the language that we had spoken about, like circling the drain, or euphemisms, or anything. Spoke very plainly and I hope clearly for the patient's family, or the patient were to be able to understand. (Man, Internal Medicine)

Interviewees identified the narrative medicine workshops as salient in prompting them to be more reflective about their own language use. One resident noted:

Definitely the language and critical illness one. I've definitely thought about it a lot actually. And even do now, when I'm in a room with a patient who's intubated and we're talking about the patient and what our plan is for them. I think it still comes to mind at times thinking about like, "Well that patient might be hearing what we're saying and this could actually be something that they remember after this too." When before I was quite, I think, just more unaware that that was something I have to think about. (Woman, Internal Medicine)

In some cases, residents described a change in themselves over the course of their intern year, such that by the end of the year they had had enough experience with language use in different clinical environments to appreciate the relevance of the workshops and the impact of dehumanizing language, especially in the critical care setting. They noted being able to separate themselves from the moment in a process of becoming self-observers reflecting on their language use. The workshops also afforded a space for higher level contemplation and introspection of not just the existence of dehumanizing language, but its impact on patients and one’s role in its propagation. As one resident reflected:

I think by the end of the year, like session six when we got to May, and we were talking about language and critical illness. And by that time I had spent time in the ICU. And actually, I was on my ICU rotation when that session happened, I thought it was actually really relevant and hit home because I had patients that were intubated, and we would talk around them and about them. And I think it definitely was very useful with pausing or allowing me to have more even self-reflection about the language I use and the things that we say around patients and their perception of it too. (Woman, Internal Medicine)

### Intent to change

Past research has shown that participants in narrative medicine curricula report developing stronger interpersonal communication skills and greater attention to their own communication practices [22]. Whether or not these trainees used language differently in actual patient care situations is not possible to verify with our data; however, in interviews some residents described that the sessions led them to modify their own language use. They noted making changes in both their written and verbal communication with an increased appreciation for how thoughts may be conveyed to both patients and colleagues.

But the language and critical illness, I definitely think that I'm much more aware of the certain phrases that are used and in trying to avoid those, especially in the ICU. (Man, Internal Medicine)

The rhetoric and discourse communities, thinking about the language that is used in one liners. And I think ever since that session, I have been very careful with specific words in the medical chart. (Woman, Internal Medicine)

I found myself changing the way I write and identify certain patients because realizing how you write certain things can really change the long-term medical treatment that they received and whether or not they're going to be targeted as, and labeled as a certain type of patient. (Man, Internal Medicine)

In these three examples, residents described how they acted on their increased awareness of the impact of language in patient care, as described above, to make changes in their practice. In addition to describing changes in their own language use, some residents shared that they felt more readily able to detect dehumanizing language use by others and empowered to correct such language use. For example, one resident imagined how they might intervene in negative language used within earshot of patients and family members:

At the very least, I feel more conscious of the type of language I'm using and that others are using around. It has not happened, but after that particular conference, I was thinking if somebody were to be using that kind of language, circling the drain, that sort of thing, loudly in areas that patients or family could hear, I might maybe pull that person aside and talk with them and say, "Hey, I get that this is the language that you may have been exposed to, but let's be more conscious of it, or at least use it when it's just us, the team around, and not people around to hear it." (Man, Internal Medicine)

Other residents reflected on the impressionable nature of medical students and identified opportunities to intervene in the socialization process, so that new trainees do not adopt dehumanizing language use as a “bad habit”:

If I hear someone say it, I do actually correct them or educate them, especially medical students who I think are learning and just keep copying words that are used by others among them who are maybe higher up and can easily fall into that bad habit. (Woman, Internal Medicine)

This sort of near peer correction is important to note, as it has been shown that the socialization that occurs within the hidden curriculum is more likely to come from fellow peers than from faculty [28]. Finally, one resident noted that they became more aware of negative language in communication with colleagues in other specialties and learned ways to reframe such conversations during the narrative medicine workshops:

I remember, later on, at some point, being on a team and thinking back to that session. It was a moment where the team that I was working on, someone, or several someones, said something about our... I think it was about our surgical colleagues that we were kind of... We get frustrated and shake our fists at our consultants and say, "Why did you do this? You're terrible." After that session, I remember being more conscious of it and speaking up and feeling good about that, just to reframe things. I had a few small phrases that I took away from that, to reframe that situation, so just saying something like, "You know, everyone is trying their best." (Woman, Internal Medicine)

Trainees who participated in the narrative medicine workshop recalled specific instances in which they not only became more conscious of dehumanizing language in healthcare, but also acted upon this awareness by trying to avoid using certain phrases in their verbal and written communication and also by challenging others who may be using such language to reconsider its use. Respondents described wanting to intervene in communication patterns so that patients and family members did not hear the medical team using dehumanizing language, correcting junior trainees so that they learned early on not to adopt prevalent dehumanizing language, and speaking up to reframe inter-specialty communication from conflict to patience. We draw particular attention to these reflections on imagined and actual courses of action, as we believe these are critically important for effecting culture change within in medical teams, health systems, and the profession at large.

## Discussion

In this research we sought to understand the effects of early exposure to a narrative medicine curriculum on internal medicine trainees’ consideration of language use in clinical care. In this qualitative study of 18 residents exploring their experience of a narrative medicine curriculum, the topic of language arose in several interviews. We identified three themes during our analysis, and found that as a result of their participation in the curriculum, residents expressed that they developed an increased awareness of the use of dehumanizing language in healthcare and out of this described a change in their own use of language both at the beside and in communication with their colleagues. Regarding our first finding of residents becoming more aware of the use of dehumanizing language, our residents commented on how the workshops helped them understand communication practices in clinical care, raised awareness of their own practices and how the culture of medicine is shaped by the use of language. Becoming aware of a situation is often the first step in addressing a problem, and our second and third findings suggest that our residents not only just developed an awareness of the use of language in the clinical context, but also moved to a process of action. At the bedside, residents recalled how what they learned in the sessions came to mind when they were caring for patients especially in critical care settings, by avoiding talking about the patient in third person and avoiding using dehumanizing phrases when speaking about patients. And lastly, some residents described speaking up or planning to speak up on their teams when negative language was used, including correcting medical students who were starting to use certain dehumanizing phrases in patient care. These findings suggest that workshops aimed specifically at addressing the use of dehumanizing language in healthcare can have meaningful impact on trainees, their patients, and colleagues and aligns with what others have found as well: that narrative medicine training in medical education can complement traditional biomedical education [29].

Research on clinicians’ language use is important because patients seeking medical care arrive with rich identities anchored in a complex network of relationships and history. Unfortunately, conventions of language use in healthcare can strip patients of their personal context and identify them solely as their disease, or worse, as a non-person. Our patient who is a 62-year-old red-haired, Catholic, sculptor grandmother of two who happens to also have type 2 diabetes, becomes “the diabetic in room four.” Or the 92-year-old retired English history teacher, who fought for his country in World War II and could beat anybody at bridge until his dementia progressed, and is now in the hospital for aspiration pneumonia, gets called a “gomer” by a tired resident. This language is then overheard by the medical student who learns that this is how “we” talk about patients. These terms can quickly become part of the jargon that is used in certain clinical situations and can have a subtle but real effect on the way we see patients. Language, and the narratives we construct with language, shape how we view and intervene in the world around us. As Donnelly writes, “case histories are not mere storage-and-retrieval devices. They are formative institutions that shape as well as reflect the thought, the talk, and the actions of trainees and their teachers” [30]. Case presentations and the language therein serve to socialize trainees into patterns of language use and patterns of thought [4]. In a team environment infused with cynicism and burnout, this may socialize trainees to use language and behave in ways that may be at odds with the goals of humanistic care. Cultural narratives therefore shape the socialization of healthcare providers and the treatment of patients.

Our work has several implications. First, residents were highly responsive to formal education about the use of language in healthcare, meaning that such education could be a ‘counter-force’ to the powerful informal curriculum where dehumanizing language is learned and the hidden curriculum that makes the use of such language appealing as a marker of professional membership. Past studies have shown that the exposure of “rude” language to patient care teams adversely affected diagnostic and procedural performance [31]. We were encouraged to find that participation in a narrative medicine curriculum motivated the residents to change their use of dehumanizing language and to intervene in others’ language use. We recommend that future work investigate the relationship between language use and clinical outcomes following narrative medicine language training.

The second implication of our study is that increasing awareness to residents’ own language use and their colleagues’ use of dehumanizing language may shed light on the larger issue of burnout. As a hallmark of burnout is depersonalization, if medical trainees are more aware of what this looks like, as expressed in language, perhaps this could enhance their ability to spot burnout in their colleagues and themselves. Professionals with high burnout show the lowest levels of empathy and conversely, the highest levels of empathy have been associated with the lowest levels of burnout, especially in the domain of depersonalization [32].

Empathy and burnout not only affect the trainee themselves but affect their patients as well, and the relationship between empathy, communication skills and patient outcomes is an important area for future research. As Neumann and colleagues argue, “Physician empathy is a particularly effective therapeutic element of patient-physician communication” and can impact patient outcomes including psychosocial outcomes as well as biomedical metrics like blood pressure and glucose control [33]. Therefore, if a trainee loses empathy, and communication skills decline, patients may be at risk for worse outcomes. In one study by Billings and colleagues, when residents were exposed to unprofessional conduct on their work teams, their own levels of burnout and cynicism rose [34]. This has also raised concerns about the ripple effect of burnout, and the relationship between burnout and empathy. Loss of empathy is a complex and nuanced phenomenon that likely has many intersecting root causes, including social support, workload, available mentorship, openness to spirituality and even choice of undergraduate major [33, 35, 36].

Another key area for future research is on the learning environment of medical training, which is the context in which language use takes place. The learning environment is a complex phenomenon, and includes not only the physical space and institutional culture, but also social dynamics, political crises, macroeconomic factors and “anything else that influences the fabric of the lives of people who engage in learning” [37]. Because medical school should be thought of as a broader learning environment, any attempts at reform must consider the informal and hidden curricula [38].

The limitations of our study suggest fruitful directions for future research. The interviews we conducted were brief and were conducted during the residents’ work hours, when they may have been under time pressure, and during the early months of a global pandemic. Additionally, our sample size is relatively small, so while our findings suggest that narrative medicine workshops can effect change in residents’ language use and promote coaching of others’ language use, the findings of this exploratory qualitative analysis, including the narrative medicine workshop as mechanism, should be further explored in future studies. While exploring residents’ language use and motivations to change language use, as we have here, is fundamentally worthy as an exploration of the hidden curriculum in medical training, these data do not allow us to verify language change or demonstrate the higher-order impacts of language use. Future research should attend specifically to demonstrating behavior change and the impact on patient outcomes of humanistic language use, attending to Kirkpatrick’s levels as a guide [39]. Additionally, we found that our residents reported most awareness of their language use in ICU settings, which may be due to the narrative medicine curriculum’s focus. Our hospital is a tertiary care hospital, and our medical ICU is one of especially high acuity. The settings in which this curriculum might come to bear with other residents at other programs would likely vary depending on acuity and structure of the services upon which they rotate. Language use is critical to the delivery of medical care in non-ICU settings, as well, and future research should investigate whether narrative medicine curricula can be used to promote changes in language use in these areas.

## Conclusion

In a learning environment where the use of dehumanizing language is pervasive, special attention must be paid to raising awareness of language use, elevating it from tacit forms of reproduction in new generations of trainees and promoting reflexivity in language use. Language use as a way to create positive organizational change has also been described by Mayfield and Mayfield in their foundational work on Motivational Language Theory (MLT) [40]. Leaders who use oral language that helps create meaning, is empathetic, and provides clear direction has been shown in the educational space to help achieve goals and objectives at the individual, collective, and organizational level [41]. Subsequent work in MLT has identified necessary antecedents, such as credibility and behavioral integrity, that should be present in leaders who hope to create durable organizational change [42]. Examining these antecedents and how they can be cultivated in medical leaders is an important focus for future work.

Given the importance of language use in clinical care and the results of our research, curricla on narrative medicine should be considered for healthcare professionals at all levels, including in faculty development. Rich opportunities exist to have this currical be interprofessional with students and faculty coming together from various disciplines with the goal of better understanding one another. Given the intersection between language use and stigma, consideration should also be given to incorporating training in narrative medicine into ongoing efforts of education in medicine on anti-bias, discrimination and micro-aggressions.

Language matters in health care, and impacts patients and providers alike. Dehumanizing language is common in health care and its use is taught and normalized through the hidden and informal curricula in medical training. Efforts to combat the use of dehumanizing language are important, as patient safety and provider burnout are at stake.

## Data Availability

The datasets used and/or analysed during the current study are available from the corresponding author on reasonable request.

## Abbreviations

ACGME: Accreditation Council on Graduate Medical Education
UME: Undergraduate Medical Education
GME: Graduate Medical education
